# Racial Disparities in Outcomes After Liver Transplantation

**DOI:** 10.7759/cureus.88693

**Published:** 2025-07-24

**Authors:** Reshad Salam, Amanda Alkhafaji, Dhruva Govil, Fred Ahmadi, Saif Affas, Mona Hassan

**Affiliations:** 1 Internal Medicine, Henry Ford Providence Hospital, Southfield, USA; 2 Gastroenterology, Henry Ford Providence Hospital, Southfield, USA; 3 Gastroenterology, University of Toledo Medical Center, Toledo, USA

**Keywords:** african american, graft survival, liver transplantation, meld score, patient survival, propensity matching, racial disparities, socioeconomic factors, transplant outcomes, unos registry

## Abstract

Racial disparities in liver transplant outcomes remain an area of concern despite advancements in organ allocation and post-transplant care. This retrospective cohort study analyzed adult liver transplant recipients from the United Network for Organ Sharing (UNOS) registry between 1988 and 2021 to evaluate differences in graft and patient survival between African American (AA) and Caucasian American (CA) recipients. After excluding non-Black and non-White individuals and pediatric cases, a 3:1 matched cohort was created using propensity-type matching for age, sex, body mass index, and ABO type, resulting in 50,584 patients (13,421 AA and 40,263 CA). Median graft survival was significantly lower in AAs compared to CAs (1,466 vs. 1,787 days, p < 0.0001), as was median patient survival (1,480 vs. 1,815 days, p < 0.0001). Graft failure rates at one year were 2,325/12,708 (18.3%) for AA vs. 5,884/37,762 (15.6%) for CA (Chi² = 50.87, df = 1, V = 0.026, p < 0.0001); at five years, 4,326/10,323 (41.9%) vs. 10,172/30,179 (33.7%), respectively (Chi² = 218.35, V = 0.060, p < 0.0001). Similarly, mortality at five years was 3,516/9,152 (38.4%) for AA vs. 8,232/26,291 (31.3%) for CA (Chi² = 154.41, V = 0.066, p < 0.0001). AAs also had higher Model for End-Stage Liver Disease (MELD) scores at listing and transplant and were more likely to be hospitalized or in the ICU at the time of transplant. Insurance coverage differed significantly, with AAs more likely to have public insurance (6,031/12,739, 47.3% vs. 13,055/36,504, 35.8%, p < 0.0001). These findings suggest that AA liver transplant recipients experience significantly worse outcomes, likely due to a combination of advanced disease at presentation and socioeconomic disparities.

## Introduction

Orthotopic liver transplantation (OLT) is the treatment of choice for patients with end-stage liver disease (ESLD). There are many individuals currently waiting for a liver transplant (LT), and the demand far outstrips the supply, with 11,101 patients on the liver candidate waiting list, with only 9,236 transplants performed in 2021 (United Network for Organ Sharing (UNOS)). In the United States, the allocation of both living and deceased liver donors is managed and guided by UNOS. The Child-Turcotte-Pugh score was a flawed scoring system used between 1996 and 2002 to allocate donor livers to recipients; however, this was replaced with the Model for End-Stage Liver Disease (MELD) score, which was more mindful of wait list mortality.

There is limited data on the influence of race on graft rejection and survival after LT [1-3]. Previous literature suggests these differences might be explained due to a variety of reasons such as the disproportionate burden of disease, differential access to care, and lower socioeconomic status [4-9]. Studies on renal transplantation have suggested African Americans (AAs) have higher graft failure rates and lower long-term survival compared to Caucasian American (CA) patients owing to possible less compliance, lower socioeconomic status, and histocompatibility mismatch [3-6].

We sought to determine if there is a difference in graft survivability, patient survival, and acute and chronic rejection rates in AA compared to CA after LT. To our knowledge, our study represents the largest study examining racial disparities in LT outcomes since the implementation of the MELD classification system.

## Materials and methods

We analyzed UNOS registry data from 1988 to 2021 for adult LT recipients, excluding pediatric cases and races other than AA and CA. The registry initially contained complete follow-up data for 326,005 patients, and after variable exclusion, it was reduced to 135,919. Using 3:1 propensity matching based on age, gender, initial body mass index (BMI), and ABO type, we created a cohort of 50,584 patients (13,421 AA and 40,263 CA).

The pool of data extracted included information on recipient age, sex, race, ABO blood group, donor type, donor age, BMI, MELD Na score at the time of listing and transplant, incidence of acute and chronic rejection, graft and patient survival time, and ICU status. Patients aged 18 years or younger were excluded, in addition to any race other than AA and CA.

Sex was divided into male or female. We classified recipient blood group types into four distinct groups: A, B, AB, and O. Donor types included either living or deceased. The percentage of individuals in each group with a BMI above 30 kg/m^2^ was reported. We examined MELD scores at the time of listing and transplant, reported as a score between 6 and 40. The percentage of patients in each race group with acute and chronic rejection was documented. Graft and patient survival time reported as the number of days since transplant was compared between the two groups.

Statistical analysis was performed using SAS 9.4 (SAS Inc., Cary, NC, US). Chi-squared tests were used for categorical comparisons; results are reported with frequency (n), percentage (%), degrees of freedom (df), Chi-squared statistic (Chi²), p-values, and Cramer's V effect size.

## Results

The data collected from UNOS included a total of 50,584 patients. Demographic and baseline clinical characteristics are detailed in Table 1. No significant differences in age, gender, or ABO were observed. Specifically, the average age of both groups was approximately 50 years, with roughly 57.2% men making up both groups. Although statistically insignificant, the proportion of obese individuals with BMI > 30 kg/m^2^ was slightly higher in the CA group (33.5% vs. 32.7%, respectively, p < 0.21). Furthermore, although there was no significant difference in donor age between the groups, there was a significant decrease in the type of donors in the CA group compared to the AA group (95.9% CA vs. 52.7% AA). The living donor type was significantly higher in the AA group compared to CA (47.3% vs. 4.1%, respectively). The ABO group distribution is similar for both AA and CA, with type A consisting of 26% vs. 25.9% (p < 0.19), respectively; type AB 5.2% vs. 5.4% (p < 0.19), respectively; type B 21% vs. 20.1% (p < 0.19), respectively; and type O 47.9% vs. 48.6% (p < 0.19), respectively. Payment type differed between the two groups, with a higher percentage of CA enrolled in private insurance compared to public insurance (64.2% vs. 35.8%, p < 0.0001) compared to AA (52.7% vs. 47.3%, p < 0.0001).

**Table 1:** Baseline demographic and clinical characteristics of liver transplant recipients by race BMI: body mass index; PELD/MELD: Pediatric End-Stage Liver Disease/Model for End-Stage Liver Disease

	Caucasian Americans (CAs) (N = 40,263)	African Americans (AAs) (N = 13,421)	p-value
Age (overall)	-	-	0.09
Mean ± SD (median)	50 ± 12 (53)	50 ± 12 (53)	-
Min to max	18 to 78	18 to 78	-
Age (category)	-	-	0.016
19-35	5,032 (12.6%)	1,799 (13.5%)	-
36-50	11,999 (30.0%)	3,904 (29.3%)	-
>50	22,989 (57.4%)	7,633 (57.2%)	-
Males	23,027 (57.2%)	7,672 (57.2%)	0.96
ABO (category)	-	-	0.19
A	10,448 (26.0%)	3,481 (25.9%)	-
AB	2,109 (5.2%)	720 (5.4%)	-
B	8,438 (21.0%)	2,701 (20.1%)	-
O	19,268 (47.9%)	6,519 (48.6%)	-
BMI (category)	-	-	0.21
<19	1,086 (2.8%)	395 (3.0%)	-
19-24.9	11,912 (30.1%)	4,000 (30.3%)	-
25-29.9	13,296 (33.6%)	4,501 (34.1%)	-
30-34.9	7,946 (20.1%)	2,624 (19.9%)	-
35+	5,280 (13.4%)	1,683 (12.8%)	-
Med Cond TRR	-	-	<0.0001
In ICU	6,503 (16.2%)	2,531 (18.9%)	-
Hospital/not ICU	7,428 (18.5%)	2,639 (19.7%)	-
Not hospitalized	26.322 (65.4%)	8,248 (61.5%)	-
Initial PELD/MELD score	N = 27,443	N = 10,309	<0.0001
Median (25th, 75th)	18 (12, 26)	20 (13, 29)	-
Initial MELD	N = 27,443	N = 10,309	<0.0001
<10	3,549 (12.9%)	1,439 (14.0%)	-
10-18	11,234 (40.9%)	3,194 (31.3%)	-
19-24	5,062 (18.5%)	2,150 (20.9%)	-
25-36	5,414 (19.7%)	2,355 (22.8%)	-
>36	2,184 (8.0%)	1,171 (11.4%)	-
MELD/PELD at time of transplant	N = 28,474	N = 10,643	<0.0001
-	21 (14, 30)	23 (15, 32)	-
MELD transplant	N = 28,474	N = 10,643	<0.0001
<10	2,800 (9.8%)	1,181 (11.1%)	-
10-18	9,152 (32.1%)	2,510 (23.6%)	-
19-24	5,644 (19.8%)	2,219 (20.9%)	-
25-36	7,630 (26.8%)	3,152 (29.6%)	-
>36	3,248 (11.4%)	1,581 (14.9%)	-
Donor type	N = 40,263	N = 13,421	<0.0001
Dead	38,621 (95.9%)	13,212 (52.7%)	-
Living	1,642 (4.1%)	6,031 (47.3%)	-
PRI_Payment_TRR cat	N = 36,504	N = 12,739	<0.0001
1: Private	23,449 (64.2%)	6,708 (52.7%)	-
2: Public	13,055 (35.8%)	6,031 (47.3%)	-
GTime	-	-	<0.0001
Median (25th, 75th)	1,787 (429, 3,728)	1,466 (377, 3,250)	-
Min to max	0 to 12,066	0 to 12,029	-
PTime	-	-	<0.0001
Median (25th, 75th)	1,815 (441, 3861)	1,480 (382, 3,278)	-
Min to max	0 to 12,066	0 to 12,029	-

MELD scores were significantly higher in AA recipients at both listing (median 20 vs. 18, p < 0.0001) and transplant (median 23 vs. 21, p < 0.0001). A higher proportion of AAs had MELD scores > 25 at transplant (4,733/10,643, 44.5% vs. 10,878/28,474, 38.2%, p < 0.0001). A higher percentage of CA had a MELD score below 19 compared to the AA group (34.7% vs. 41.9%, respectively, p < 0.0001). Table 1 also shows that a higher proportion of CA compared to AA are not hospitalized before undergoing LT (65.4% vs. 61.5%, p < 0.0001). AA had higher ICU admission rates at the time of transplant (2,531/13,421, 18.9% vs. 6,503/40,263, 16.2%, p < 0.0001) and were less likely to have private insurance (6,708/12,739, 52.7% vs. 23,449/36,504, 64.2%, p < 0.0001). The proportion of hospitalized patients not in the ICU was slightly higher in the AA group compared to CA (18.5% vs. 19.7%, respectively, p < 0.0001).

**Figure 1:**
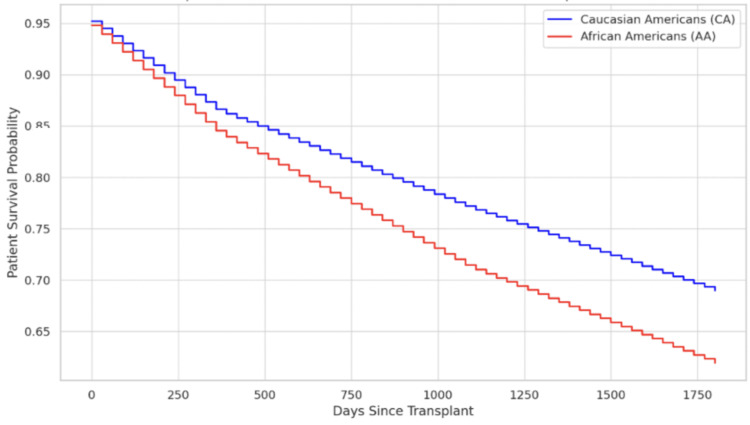
Kaplan-Meier plot displaying declining graft survival over 5 years. As seen, there is a consistently lower curve for AA recipients

Table 1 shows that the graft survival time was significantly higher in CA with a median survival time of 1,787 days compared to 1,466 days in AA (p < 0.0001), adjusted for age, gender, BMI, and ABO type. Furthermore, graft failure rates were significantly higher in the AA population compared to CA. Table 2 demonstrates graft failure rates between CA and AA on day 30 (6.5% vs. 7.3%, p = 0.0009), one year (15.6% vs. 18.3%, p < 0.0001), three years (25.4% vs. 32.1%, p < 0.0001), and five years (33.7% vs. 41.9%, p < 0.0001), adjusted for age, gender, BMI, and ABO type. This resulted in an increasing gap between the two racial groups on the Kaplan-Meier plot for graft survival time, with AA having a poorer graft survival rate compared to CA at five-year post-OLT (Figure 1).

**Table 2:** Graft failure rates by race and time. This shows that AAs have significantly higher rates at every time point

	Caucasian Americans (CAs) (N = 40,263)	African Americans (AAs) (N = 13,421)	p-value	Chi-squared test (Chi²)	Degrees of freedom (df)	Cramer’s effect size (V)
Gfail day 30	2,568/39,584 (6.5%)	970/13,245 (7.3%)	0.0015	10.05	1	0.014
Gfail 1 year	5,884/37,762 (15.6%)	2,325/12,708 (18.3%)	<0.0001	50.87	1	0.026
Gfail 3 years	8,426/33,150 (25.4%)	3,625/11,310 (32.1%)	<0.0001	154.95	1	0.050
Gfail 5 years	10,172/30,179 (33.7%)	4,326/10,323 (41.9%)	<0.0001	218.35	1	0.060

Additionally, patient survival time was significantly higher for CA with a median time of 1,815 days as opposed to 1,480 days in the AA cohort (Table 1). Table 3 demonstrates patient survival rates at day 30 (4.8% vs. 5.2%, p = 0.059), one year (13.5% vs. 15.6%, p < 0.0001), two years (23% vs. 28.8%, p < 0.0001), and five years (31.3% vs. 38.4%, p < 0.0001).

**Table 3:** Patient mortality differences based on race and time

	Caucasian Americans (CAs) (N = 40,263)	African Americans (AAs) (N =1 3,421)	p-value	Chi-squared test (Chi²)	Degrees of freedom (df)	Cramer’s effect size (V)
Died day 30	1,703/35,493 (4.8%)	640/12,248 (5.2%)	0.0625	3.47	1	0.009
Died 1 year	4,404/32,753 (13.5%)	1,772/11,396 (15.6%)	<0.0001	30.91	1	0.026
Died 3 years	6,642/28,855 (23.0%)	2,908/10,111 (28.8%)	<0.0001	133.13	1	0.058
Died 5 years	8,232/26,291 (31.3%)	3,516/9,152 (38.4%)	<0.0001	154.41	1	0.066

## Discussion

People of color are projected to make up roughly 52% of the population in the United States by 2050 [10]; therefore, it is important to explore disparities in healthcare faced by different racial/ethnic groups. Racial disparities in health remain an area of concern, and the impact of race on solid organ transplantation outcomes has been previously established in renal studies [11,12]. These studies have identified several contributing factors for differences in outcomes such as low referral rates, poor human leukocyte antigen (HLA) matching, severity of illness, comorbidities, non-compliance, low socioeconomic status, and health inequity [11-16]. There is conflicting data in LT studies with no clear consensus on the differences between racial differences in outcomes [17-21]. Although advancements in immunosuppression, surgical techniques, organ allocation, and patient selection have improved patient survival, there is evidence of racial disparities in LT outcomes [1-3].

Our study demonstrated that race is an independent factor of graft and patient survival time. These disparities grow more pronounced over time, as demonstrated by widening survival curves and escalating differences in graft failure and mortality at three and five years. To our knowledge, this is the largest study investigating the racial disparities in outcomes after primary LT. Table 2 highlights the difference in graft failure rates between the two groups and demonstrates that the AA group has a consistently higher graft failure rate compared to CA during all interval periods. Our propensity-matched, adjusted data show that compared to CAs, AAs have a 6.7% and 8.2% higher risk of three-year and five-year post-LT graft failure, respectively (p < 0.0001); however, this difference is modest at day 30 and one year post-LT. This is reflected in the Kaplan-Meier curve plotting graft survival rate (Figure 1), which shows a widening gap between the two groups over time and a lower survival rate in the AA group compared to CA over a five-year period. Table 3 shows a significant difference in patient survival rates between the two groups, with AA having consistently lower patient survival rates. AA had a 5.8% and 7.1% lower survival rate compared to CA at three and five years post-LT (p < 0.0001), but the difference is insubstantial for 30-day and one-year survival rates.

The effect of age on patient and graft survival has been established in previous studies, with younger patients enjoying a more favorable outcome [3]. However, when we look at our data, the population of the two groups is on average the same age, and both groups have comparable age range percentages (Table 1). We believe one of the reasons why graft and patient survival times differed between the two groups is likely due to AA initially presenting at a severe stage of liver failure. A study by Reid et al. showed that AAs had more severe liver disease at the time of listing and were more likely to die while waiting compared to CA [22]. This is reflected in our data that show that the initial MELD score is higher in the AA group for the scores in the upper ranges (initial MELD > 20) ranging between 19 and 24 (18.5% vs. 20.9%, p < 0.0001) and 25 and 36 (19.7% vs. 22.8%, p < 0.0001) and initial MELD greater than 36 (8% vs. 11.4%, p < 0.0001) (Table 1). Similarly, the MELD score at the time of transplant is greater in the AA group with a 7.4% higher chance of having a MELD score of 19 or higher (p < 0.0001). Nair et al. reported higher severity of liver disease at the time of presentation in addition to worse survival time at day 30 in AA compared to CA [1]. This is not seen in our data set, however, and would indicate that there are other potential contributing factors affecting survival outcomes. Our data did, however, establish that the proportion of hospitalized and ICU patients was significantly higher in AA compared to CA, with a 2.7% higher chance of AA being in the ICU and a 1.2% higher chance of being hospitalized but not in the ICU compared to CA. These findings suggest AAs are generally sicker, require higher acuity medical management at the time of LT, and are at a more advanced stage of liver disease compared to CA. This likely explains the worst patient and graft survival time as described earlier.

The reasons for the study differences are likely multifactorial in nature. Usually, AA patients present with a more severe liver disease, have higher ICU admissions, and have a greater reliance on public insurance. Specifically, disparities in the timely management of liver disease in the AA population have been reported to stem from socioeconomic differences in education level, insurance type, income, and access to healthcare [23,24]. Our study showed a significantly higher percentage of CA having private insurance compared to AA (64.2% vs. 52.7%, p < 0.0001). This could possibly be a contributing factor to the difference in graft, patient survival times, and rejection rates. A previous study by Yoo and Thuluvath reported similarly that patients with public insurance had lower overall patient survival at two years [4]. Similarly, in a study by Ananthakrishnan and Saeian, public insurance was found to be associated with worse patient survival and graft survival in AA compared to CA at two years post-LT with HR 1.16, 95% CI 1.06-1.27 and HR 1.22, 95% CI 1.10-1.40, respectively [2]. The graft and patient survival time in our study, unfortunately, could not be adjusted for insurance type, as there was insufficient data. We could, however, make the case that insurance type as a medium of socioeconomic status continues to influence LT outcomes in the MELD era, as was seen in the pre-MELD period. Our study was unable to assess other socioeconomic contributing factors and substantiate their effect due to the lack of data in the national transplant registries. HLA mismatch and higher rejection rates have been presented in previous studies on solid organ transplantation as probable factors affecting graft and patient survival time [1,25,26]. Our study did not include HLA mismatch data as it was not available; however, analysis of ABO distribution in our data showed a similar spread of blood types A, AB, B, and O among the two racial groups (Table 1). Differences in response to immunosuppression and underlying comorbidities are also mentioned in the literature as potential causes influencing mortality and rejection rates [1,27]. Higher rates of acute and chronic rejection in AA have been reported in previous studies, with heightened immunoresponsiveness increasing the risk of rejection in AA [28]. In a study by Nagashima et al., AA patients undergoing tacrolimus-based immunosuppression regimen post-LT were found to have decreased immunosuppression compared to CA, with a higher risk of acute and chronic graft rejection [29]. The etiology of liver disease has been shown to be a significant factor in the mortality rate in AA, with hepatitis C and hepatocellular carcinoma being associated with a higher mortality rate compared to CA [2].

The data we analyzed lack information on liver disease etiology, and further research is needed to establish if this is a contributing factor, in addition to the burden of comorbid illness. Furthermore, our study could be expanded to look at other sub-groups, such as Hispanics and Asians. Our limitations also include a lack of data on comorbid conditions, etiology of liver disease, socioeconomic status, compliance, and type of immunosuppression. Yet, the study's large sample size, matched cohorts, and rigorous statistical reporting reinforce the validity of our findings when comparing the CA and AA groups.

Though early outcomes (30-day mortality) showed no statistical difference, longer-term outcomes were consistently worse in AAs. The modest but consistent effect sizes (Cramer's V ranging 0.026-0.066) highlight the clinical relevance of these racial differences. To conclude, our study has shown a significant difference in patient and graft survival time in AA compared to CA after OLT. The reasons for these racial differences are likely multifactorial, and further studies are necessary to establish contributing factors.

## Conclusions

This large, national analysis of adult LT recipients demonstrates a persistent and statistically significant disparity in both graft and patient survival between AA and CA. Despite similar age, gender, and ABO matching, AA experienced higher MELD scores at transplant, more frequent ICU admissions, and greater graft failure and mortality rates at every follow-up interval beyond 30 days. These differences likely stem from multifactorial contributors, including advanced disease severity at presentation, differences in access to care, insurance status, and potential immunologic and biologic factors. While the MELD system aimed to improve equity in organ allocation, our findings suggest that systemic disparities in LT persist. Further research incorporating disease etiology, HLA mismatch, immunosuppression adherence, and socioeconomic data is needed to fully understand and address the underlying causes. Targeted interventions are critical to improve equity and outcomes in LT for AA patients.
